# Patients with Primary Open-Angle Glaucoma May Develop Ischemic Heart Disease More Often than Those without Glaucoma: An 11-Year Population-Based Cohort Study

**DOI:** 10.1371/journal.pone.0163210

**Published:** 2016-09-20

**Authors:** Yu-Yen Chen, Hsiao-Yun Hu, Dachen Chu, Hsin-Hua Chen, Chin-Kuo Chang, Pesus Chou

**Affiliations:** 1 School of Medicine, National Yang-Ming University, Taipei, Taiwan; 2 Department of Ophthalmology, National Yang-Ming University Hospital, Yilan, Taiwan; 3 Department of Education and Research, Taipei City Hospital, Taipei, Taiwan; 4 Community Medicine Research Center and Institute of Public Health, National Yang-Ming University, Taipei, Taiwan; 5 Deputy Superintendent, Taipei City Hospital, Taipei, Taiwan; 6 Department of Medical Research, Taichung Veterans General Hospital, Taichung, Taiwan; 7 Institute of Biomedical Science and Rong Hsing Research Center for Translational Medicine, Chung-Hsing University, Taichung, Taiwan; 8 Division of Allergy, Immunology and Rheumatology, Department of Internal Medicine, Taichung Veterans General Hospital, Taichung, Taiwan; 9 School of Medicine, Chung-Shan Medical University, Taichung, Taiwan; 10 Department of Medical Education, Taichung Veterans General Hospital, Taichung, Taiwan; 11 Department of Psychological Medicine, Institute of Psychiatry, Psychology and Neuroscience, King’s College London, London, United Kingdom; Indiana University, UNITED STATES

## Abstract

**Objectives:**

To investigate whether patients with primary open angle glaucoma (POAG) have a higher proportion of ischemic heart disease (IHD) development.

**Design:**

A population-based retrospective cohort study, using the National Health Insurance Database (NHID) from 1^st^ January, 2001, to 31^st^ December, 2011, in Taiwan.

**Methods:**

3510 subjects with POAG were enrolled into the POAG group and 14040 subjects without glaucoma into the comparison group. The comparison group consisted of randomly selected individuals, matched with the POAG group based on age, gender, and index date (date of enrollment) at a ratio of 1:4. The participants of both groups should have no IHD before the index date, and they were followed until the end of 2011 to see whether they had new-onset IHD or not. Kaplan-Meier curves were used to compare the cumulative incidence of IHD between the two groups. Frailty model, a specialized form of Cox regression analysis, was used to estimate the crude and adjusted hazard ratio (HR) of IHD. Analyses were adjusted by age, gender, and systemic comorbidities (i.e. diabetes, hypertension, hyperlipidemia, atrial fibrillation and congestive heart failure).

**Results:**

The mean age of the cohort was 57.6±11.0 years. There were slightly more males than females (51.6% vs. 48.4%). A log-rank test comparing Kaplan-Meier curves of the two groups revealed a significantly higher cumulative incidence of IHD in the POAG group (*p*-value<0.001). In the univariate analysis by Frailty model, POAG patients had a significantly higher hazard of IHD (unadjusted HR = 2.32; 95% confidence interval 1.93 to 2.79). After adjustment, results remained significant (adjusted HR = 1.41; 95% confidence interval 1.16 to 1.72).

**Conclusion:**

People with POAG may suffer from IHD more often than those without glaucoma.

## Introduction

Glaucoma is the leading cause of irreversible blindness worldwide. [1] Previous studies predict that there will be over 75 million glaucoma patients in 2020, [1,2] increasing to over 110 million in 2040. [2] Of these cases, 74% will be open-angle glaucoma. [1] Primary open-angle glaucoma (POAG) is a progressive, chronic optic neuropathy in adults, with characteristic optic nerve fibers damage and associated visual field loss. [3] The term “open” is used in reference to an open anterior chamber angle and “primary” in reference to an absence of secondary etiologies (e.g., uveitis, trauma, corticosteroid use).

Although elevated intraocular pressure (IOP) is the most common and the only modifiable risk factor of POAG, there are many other factors that can contribute to optic nerve damage or disease progression. [4–8] One hypothetical cause may be insufficient ocular blood flow leading to optic nerve ischemia and glaucomatous optic neuropathy. [6,9–11] In animal models, diminished ocular perfusion has been shown to induce retinal ganglion cell loss in spite of a normal IOP. [12,13] In humans, hospital-based studies have also reported blood flow deficiency or instability in POAG patients. An insufficiency in blood flow has been reported to occur in retina, [14] choroid [14,15] and retrobulbar areas. [16]

Based on this vascular theory hypothesis of POAG, large epidemiologic studies have been performed to investigate the relationship between cardiovascular conditions and POAG. Bonomi *et al*. reported an association between lower diastolic perfusion pressure and POAG in the Egna-Neumarkt Study. [17] Similarly, the Singapore Malay Eye Study found a significant association between lower diastolic blood pressure and lower diastolic perfusion pressure and open angle glaucoma. [18] The Early Manifest Glaucoma Trial (EMGT) also found reductions in ocular perfusion pressure to be related to the progression of glaucoma. [19]

In the 1920s, glaucomatous eye was first referred to as “a sick eye in a sick body”. [20,21] Flammer J and Flammer AJ, in a reviewed article published in 2013, furtherly addressed the concept that vascular changes in the eye may be an early indicator of heart diseases because the two organs share many common characteristics and expose to the same intrinsic and environmental influences. [22] Furthermore, POAG patients were found to have characteristics of atherosclerosis, autonomic dysfunction and endothelial dysfunction, which may lead to diminished ocular blood flow. [23–25] These characteristics may also influence the vasculature of the heart and decrease blood flow to the myocardium, leading to ischemic heart disease (IHD). [26] Thus, we hypothesized the incidence of IHD would be higher in POAG group than non-POAG group. Previous studies, using 24-hour ECG monitoring, found patients with POAG had higher frequencies of silent myocardial ischemia and significant asymptomatic ST-T segment depression. [27,28] However, Gherghel et al. found that although the autonomic function in patients with POAG was different from healthy subjects, there was no significant difference in silent cardiac ischemic episodes between the two groups. [29] The inconsistent findings of these investigations may be due to the fact that they only studied a small number of cases. Therefore, we conducted a population-based follow-up cohort study to explicitly investigate whether patients with POAG suffer from IHD more often than those without glaucoma.

## Materials and Methods

### Study setting

Taiwan’s National Health Insurance (NHI) program, which was launched in 1995, currently covers the health care services of more than 98% of Taiwan’s 23 million residents. [30] The National Health Insurance Research Database (NHIRD), which is maintained by Taiwan’s National Health Research Institutes, is a collection of all registration file data and claims data for all of Taiwan’s ambulatory and in-hospital patients. The identification of all patients in the database is encrypted before the data is released. [31] We used a subset of this database, known as the Longitudinal Health Insurance Database, to perform this study. According National Health Research Institutes, there is no significant age, gender, or healthcare cost difference between this randomly selected subset and all enrollees. [31] This study was approved by the ethical committee of Yang-Ming University Hospital (2015A018). Each patient record was anonymized and de-identified prior to analysis, therefore the informed consent was exempt from review according to the Institutional review board.

### Study sample

Using Taiwan NHI’s well-characterized longitudinal database, we performed a retrospective cohort study with a follow-up period from January 1, 2001 to December 31, 2011. We first selected patients with a diagnosis of primary open-angle glaucoma (POAG) identified on claims records using the International Classification of Diseases, 9^th^ Revision, Clinical Modification Codes (ICD-9-CM code: 365.11) during study period. To focus our study on the relationship of POAG and IHD, those who had ever had the diagnosis of exfoliative glaucoma or secondary glaucoma were excluded from our study sample. To assure the validity of the diagnoses, only patients who had at least three times of POAG diagnoses during the period were selected into POAG group. Date of first POAG diagnosis was defined as the index date. Those who had never received antiglaucoma drugs or glaucoma surgeries, and those who were younger than 40 years old were excluded. We also randomly selected individuals who had never received a diagnosis of any type of glaucoma (ICD-9-CM codes: 365.X) as a comparison group at a ratio of 1:4, matched with the study group on age, gender, and index year (the year of index date). Outcome variable was IHD, which included acute, subacute, chronic myocardial infarction, and angina pectoris (ICD-9-CM codes: 410–411, 413–414). To confirm the IHD diagnoses, IHD patients should have at least three times of IHD diagnoses. They had received standard protocols of examinations, including history taking, physical examination, biochemical test, electrocardiography, and/or image (e.g., echocardiography, coronary angiography, cardiac magnetic resonance, nuclear perfusion scan, cardiac positron emission tomography). All eligible POAG subjects and comparison individuals were assured to have not ever received a diagnosis of IHD before the index date.

### Covariates

The covariates we adjusted for were age and gender as well as risk factors for IHD, including diabetes (ICD-9-CM code:250), hypertension (ICD-9-CM code:401–405), hyperlipidemia (ICD-9-CM code:272), atrial fibrillation (ICD-9-CM code:423.71), and congestive heart failure (ICD-9-CM code:428). [32] These comorbidities were identified in the medical records and were regarded as potential confounders.

### Statistical analysis

After investigating the two groups descriptively by age, gender, and comorbidity, the group differences were analyzed by two-sample *t*-test (for continuous variables) and qi-square test (for categorical variables). Survival analysis using Kaplan-Meier method with a log-rank test was applied to describe and compare the cumulative incidence curves of IHD. Frailty model, [33,34] a specialized form of Cox proportional hazard model, was used to estimate the hazard ratio (HR) for the occurrence of IHD according to each variable in univariate and multivariate analyses. Variables included in the regression analysis were age, gender and relevant comorbidities, including diabetes, hypertension, hyperlipidemia, atrial fibrillation, and congestive heart failure. Comorbidities were regarded as time-dependent covariates. All statistical operations were performed using SAS statistical package, version 9.2 (SAS Institute, Cary, NC, USA).

## Results

### Demographic characteristics of the study sample

This study included a total of 3510 POAG patients and 14040 matched comparisons. Table 1 is a summary of the characteristics of the two groups. Because the two groups were matched on age and gender, there was no difference in these two variables. The mean age was 56.7 years. Nearly 40 percent were over 60 years old. Males made up a slightly higher proportion than females (51.6% vs. 48.4%). The POAG group had higher percentage of diabetes, hypertension, hyperlipidemia, congestive heart failure and atrial fibrillation than the comparison group.

**Table 1:** Characteristics of study subjects.

Variable	POAG group	Comparison Group	*p*-value
	*n* = 3,510	*n* = 14,040	
	n (%)	n (%)	
**Age**,year, (mean±SD)	56.7±11.0	56.7±11.0	1.000
40–50	1096 (31.2)	4384 (31.2)	1.000
50–60	1081 (30.8)	4324 (30.8)	.
60–70	880 (25.1)	3520 (25.1)	.
≥70	453 (12.9)	1812(12.9)	.
**Gender**			1.000
Male	1812 (51.6)	7248 (51.6)	
Female	1698 (48.4)	6792 (48.4)	.
**Comorbidities**			
Diabetes			<0.0001
Yes	1208 (34.4)	2568 (18.3)	
No	2302 (65.6)	11472 (81.7)	
Hypertension			<0.0001
Yes	1894 (54.0)	5252 (37.4)	
No	1616 (46.0)	8788 (62.6)	
Hyperlipidemia			<0.0001
Yes	1358 (38.7)	3590 (25.6)	
No	2152 (61.3)	10450 (74.4)	
Congestive heart failure			
Yes	184 (5.2)	638 (4.5)	0.08
No	3326 (94.8)	13402 (95.5)	
Atrial fibrillation			
Yes	60 (1.7)	186 (1.3)	0.09
No	3450 (98.3)	13854 (98.7)	

POAG: Primary open angle glaucoma

### Cumulative incidence curves by the Kaplan-Meier method

**Fig 1:**
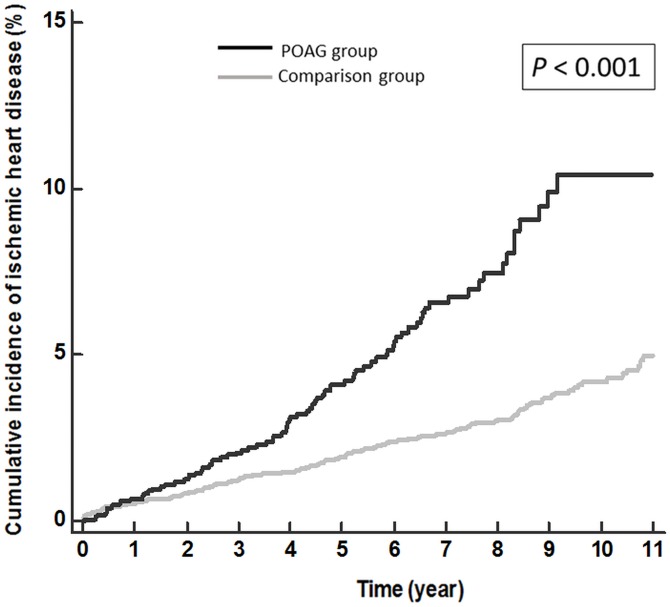
Kaplan-Meier curves for IHD among POAG and comparison groups. Black line represents POAG group and gray line represents comparison group.

Fig 1 illustrates the cumulative incidence curves of IHD in POAG group and comparison group analyzed using the Kaplan-Meier method for describing stratified time-to-event data. From the very beginning of follow up, these two curves were constantly moving apart from each other till the end. A log rank test comparing these two curves revealed a statistically significant difference (log rank, *p*-value < 0.001).

### Univariate and multivariate analyses by Cox models

Table 2 shows the results of our frailty model. The unadjusted hazard of IHD in the POAG group was 2.32 times that of the comparison group, with a 95% confidence interval (CI) of 1.93 to 2.79. After adjustment of other factors, POAG group still had a significantly higher hazard of IHD (adjusted HR = 1.41, 95%CI 1.16 to 1.72). Age was also a significant risk factor for IHD in both univariate and multivariate analyses. The adjusted HR of IHD in patients over 70 years old reached 2.97 when compared with those between 40 to 50 years old. Men were more likely to develop IHD than women (unadjusted HR = 1.50 with 95% CI 1.26 to 1.79, adjusted HR = 1.61 with 95% CI 1.35 to 1.93). All people with the comorbidities we studied (diabetes, hypertension, hyperlipidemia, congestive heart failure, atrial fibrillation) had significantly increased hazard ratio for IHD.

**Table 2:** Analyses of Risk Factors for IHD in Patients with and without POAG.

	Univariate analysis		Multivariable analysis	
Predictive variables	Unadjusted HR	*P* value	Adjusted HR	*P* value
	(95% CI)		(95% CI)	
**POAG** (Yes v.s. No)	2.32(1.93–2.79)	<0.0001	1.41(1.16–1.72)	0.0006
**Age**				
40–50	Reference		Reference	
50–60	2.84(2.04–3.95)	<0.0001	2.14(1.53–2.99)	<0.0001
60–70	4.45(3.24–6.11)	<0.0001	2.63(1.90–3.64)	<0.0001
≥70	5.86(4.23–8.12)	<0.0001	2.97(2.11–4.16)	<0.0001
**Gender** (Male)	1.50(1.26–1.79)	<0.0001	1.61(1.35–1.93)	<0.0001
**Comorbidities** (Yes v.s. No)				
Diabetes	3.65(3.05–4.36)	<0.0001	1.83(1.50–2.23)	<0.0001
Hypertension	2.46(2.03–2.99)	<0.0001	2.11(1.68–2.64)	<0.0001
Hyperlipidemia	3.07(2.57–3.68)	<0.0001	1.50(1.22–1.83)	0.0001
Congestive heart failure	8.56(6.89–10.63)	<0.0001	4.09(3.24–5.17)	<0.0001
Atrial fibrillation	5.94(4.04–8.76)	<0.0001	1.94(1.29–2.92)	0.0014

HR indicates hazard ratio; CI indicates confidence interval

IHD: ischemic heart disease; POAG: Primary open angle glaucoma

All factors with p < 0.1 in univariate analyses were included in the Cox multivariate analysis.

## Discussion

### Main findings

In this 11-year follow-up study using nation-wide, population-based data, we found that patients with POAG had a higher cumulative incidence of IHD during the follow-up period and that their hazard for IHD was 40 percent higher than those without POAG. Older age, male gender, and the comorbidities (diabetes, hypertension, congestive heart failure, atrial fibrillation, hyperlipidemia) also increased the possibilities of developing IHD.

### Comparisons with related studies

In addition to our main findings, we also found that a higher percentage of patients with POAG had diabetes, hypertension, congestive heart failure, atrial fibrillation, and hyperlipidemia than the comparison group. Similarly, Lin HC et al, using a single year of NHIRD data to compare the prevalence of systemic diseases in open-angle glaucoma (OAG) group and non-OAG group, [35] found the prevalence for the following diseases: diabetes 30.2% vs. 19.5%, hypertension 50.5% vs. 40.3%, congestive heart failure 6.5% vs. 5.0%, and hyperlipidemia 30.5% vs. 19.3%. Their study also revealed that the OAG group had a significantly higher prevalence of IHD than the comparison group (2.4% vs. 1.9%). Our study, based on long-term longitudinal data furtherly revealed POAG group had a significant higher incidence and hazard of IHD.

To the best of our knowledge, only a few studies have specifically evaluated the relationship between POAG and IHD. These studies utilized 12-lead echocardiogram (ECG) to detect the ischemic changes of cardiac muscle. [27–29] Kaiser et al. found one of seven (14.3%) patients with POAG had silent myocardial ischemia (MI), not exceeding the proportion of silent MI in patients with cataracts (3 of 20, 15.0%). [27] In a paper published in 2007 comparing 23 glaucoma patients and 22 control subjects, Gherghel et al. reported the two groups to have similar prevalence of IHD. [29] In contrast, Waldmann et al. found 7 of the 27 (25.9%) POAG patients to have silent MI, which was higher than the proportion of silent MI in either cataract patients (3 of 25, 12%) or normal controls (1 of 20, 5%). [28] Perasalo et al., investigating 213 institutionalized geriatric glaucoma patients and 100 control patients, [36] also found that IHD occurred more frequently in geriatric glaucoma patients than in their control group (56% vs. 38%). In sum and in short, these previous studies were hospital-based investigations with small case numbers, and did not include some important confounders into their analyses. In addition, their diagnosis of IHD was based on ECG changes alone without confirmation of other examinations. In our study, IHD patients should have at least three times of IHD diagnoses. And, in our health-care system, patients have to receive standard protocols of examinations to confirm the diagnosis, or the National Health Insurance Administration will not pay the fees for treatment. Therefore, the diagnoses in our study, including the diagnoses of POAG and comorbidities, were highly verified. Besides, our population-based study analyzed nationwide dataset and could achieve high representativeness and high power. In our analysis, we included age, gender, and some systemic comorbidities as confounders because they were risk factors for IHD. With the adjustment of confounders, we can derive a more real relationship between POAG and IHD. Furthermore, our long-term follow-up cohort study, different from cross-sectional design of previous studies, could provide more concepts about incidence and risk factors of IHD.

### Biomedical explanations

Emre et al. demonstrated that ocular blood flow alteration in glaucoma patients is related to systemic vascular dysregulation. [37] Previous literature revealed that the underlying mechanisms of vascular dysregulation include atherosclerosis, dysfunction of autonomic nervous system, and vascular endothelial cells dysfunction. [6,24] Ronkko et al. found that in the ocular tissue of OAG patients, there is over-expression of a specific enzyme which indicates atherosclerosis. [25] Some previous studies have also found that POAG patients exhibit autonomic dysfunction, [24,38,39] Another mechanism of vascular dysregulation is endothelial dysfunction. Plasma endothelin-1 (ET-1), a very potent vasoconstrictor produced mainly by vascular endothelial cells, has been found to be increased in glaucoma patients. [23]. These underlying mechanisms of vascular dysregulation in POAG may all together lead to perfusion instability and ischemic changes. [40]

The ischemic changes in IHD are similar to those of POAG. The most common cause of IHD is atherosclerosis of the coronary arteries. [26] Dysregulation in autonomic nervous system is an important trigger of coronary vasospasm, [41,42]which is often called Prinzmetal’s angina. [43]

Other possible triggers include ET-1, which has been found to be elevated both in POAG and in IHD patients. [44] In summary, According to previous literature, POAG and IHD, in the perspective of hemodynamics regarding vascular dysregulation, may have similar or share common pathogenesis (e.g., atherosclerosis, dysfunction of autonomous nervous system, endothelial dysfunction) and may explain the reason that this study found that patients with POAG suffer from IHD more often.

In our study, we utilized National-Health Insurance Database, which lacks laboratory data. Thus, our focus is on presenting the higher proportions of developing IHD among POAG patients based on our database. It is beyond the scope of our study to prove the biomedical explanations. Further advanced laboratory studies will be need to explore the underlying mechanisms and the definite explanations.

### Limitations

Our study has some limitations. Some of the individuals in the comparison group may have undiagnosed POAG. According to previous studies, the prevalence of glaucoma in Asians ranges from 2.1% to 5.0%, [45] and approximately 50% of glaucoma cases are undetected. [46] Together, these statistics suggest that 1.05% to 2.5% of the population has undetected glaucoma. In our study, subjects with undetected glaucoma were classified as comparison group, not POAG group. Such a misclassification bias would weaken the effect of POAG on the development of IHD. Even so, POAG patients in our study had a higher incidence and hazard of IHD. Therefore, our observation of an increased IHD hazard in the POAG group is a real phenomenon.

Another limitation is that data on smoking, obesity, and substance use, which might contribute to the development of IHD, [47–49] were not available in our database. In statistics, a confounder is a variable that is associated with both dependent variable (e.g., IHD in our study) and independent variable (e.g., POAG in our study). [50] Since most studies have revealed smoking is not associated with POAG, [51–55] smoking is not a confounder in our study. Similarly, some studies have revealed obesity is not associated POAG, [54,56] inferring that obesity will not confound our results. However, substance use disorder has been proposed to be associated with open-angle glaucoma. [57] Therefore, substance use is a confounder in our study. Unfortunately, NHIRD did not supply detailed data about substance use. Further epidemiologic studies will be needed to provide such information. Still another limitation is the central neurological pressure, which is recently found to be related to the development of glaucoma, [58–61] is not included in our study. Further clinical or laboratory studies will be needed to address this issue.

It is noteworthy that in the field of glaucoma with a strict definition, POAG includes high tension glaucoma (HTG) and normal tension glaucoma (NTG). However, in the definition of ICD-9-CM codes, POAG and NTG had different codes (365.11 and 365.12, respectively). Thus, POAG (365.11) in ICD-9-CM codes means HTG. Previous studies revealed the vascular risk factors are not exactly the same between HTG and NTG. [62,63] In order to derive a clarified relationship in our study, we merely focus on those with the ICD-9-CM code 365.11 (actually, HTG). We will conduct further study to evaluate the association between NTG (ICD-9-CM code: 365.12) and IHD. As far as HTG and NTG are concerned, they appear to be a continuum of glaucomas, in which the underlying mechanisms shifts from predominantly elevated IOP in HTG to hemodynamic change in NTG. In other words, HTG and NTG both are related to hemodynamics, but there are more hemodynamic-related properties in NTG. If the association between HTG and IHD has been found to be significant, a stronger association is likely to be presented between NTG and IHD.

### Clinical implications and conclusion

Our study revealed that POAG patients may suffer from IHD more often than those without glaucoma. We have to reiterate that we did not conclude POAG is a risk factor of IHD or any causal relationship between these two diseases. What we present is, based on the analysis of our database, a higher proportion of IHD development among POAG patients. This may be due to the similar underlying mechanisms of the two diseases. Further research should be conducted to help validate and clarify the underlying pathophysiological mechanisms as well as the definite biomedical explanations. And, ophthalmologists are suggested to pay more attention to the cardiovascular disease when treating POAG patients.
